# Prognostic Impact of Modified J‐MACS Score in Patients With Systolic Heart Failure Receiving Transcatheter Edge‐to‐Edge Mitral Valve Repair

**DOI:** 10.1161/JAHA.125.043819

**Published:** 2025-09-30

**Authors:** Yohei Ueno, Teruhiko Imamura, Shuhei Tanaka, Hiroshi Ueno, Koichiro Kinugawa, Shunsuke Kubo, Masanori Yamamoto, Yuki Izumi, Mike Saji, Masahiko Asami, Yusuke Enta, Shinichi Shirai, Masaki Izumo, Shingo Mizuno, Yusuke Watanabe, Makoto Amaki, Kazuhisa Kodama, Hisao Otsuki, Toru Naganuma, Hiroki Bota, Yohei Ohno, Daisuke Hachinohe, Masahiro Yamawaki, Gaku Nakazawa, Toshiaki Otsuka, Kentaro Hayashida, OCEAN‐Mitral investigators

**Affiliations:** ^1^ Second Department of Internal Medicine University of Toyama Toyama Japan; ^2^ Department of Cardiology Kurashiki Central Hospital Kurashiki Japan; ^3^ Department of Cardiology Toyohashi Heart Center Toyohashi Japan; ^4^ Department of Cardiology Nagoya Heart Center Nagoya Japan; ^5^ Department of Cardiology Gifu Heart Center Gifu Japan; ^6^ Department of Cardiology Sakakibara Heart Institute Tokyo Japan; ^7^ Division of Cardiovascular Medicine, Department of Internal Medicine Toho University Faculty of Medicine Tokyo Japan; ^8^ Division of Cardiology Mitsui Memorial Hospital Tokyo Japan; ^9^ Department of Cardiology Sendai Kosei Hospital Sendai Japan; ^10^ Division of Cardiology Kokura Memorial Hospital Kitakyushu Japan; ^11^ Division of Cardiology St. Marianna University School of Medicine Hospital Kawasaki Japan; ^12^ Department of Cardiology Shonan Kamakura General Hospital Kanagawa Japan; ^13^ Department of Cardiology Teikyo University School of Medicine Tokyo Japan; ^14^ Department of Cardiology National Cerebral and Cardiovascular Center Suita Japan; ^15^ Division of Cardiology, Saiseikai Kumamoto Hospital Cardiovascular Center Kumamoto Japan; ^16^ Department of Cardiology Tokyo Woman’s Medical University Tokyo Japan; ^17^ Department of Cardiology New Tokyo Hospital Chiba Japan; ^18^ Department of Cardiology Sapporo Higashi Tokushukai Hospital Sapporo Japan; ^19^ Department of Cardiology Tokai University School of Medicine Isehara Japan; ^20^ Department of Cardiology, Sapporo Heart Center, Sapporo Cardiovascular Clinic Sapporo Japan; ^21^ Department of Cardiology Saiseikai Yokohama City Eastern Hospital Kanagawa Japan; ^22^ Division of Cardiology, Department of Medicine Kinki University Faculty of Medicine Osaka Japan; ^23^ Department of Hygiene and Public Health Nippon Medical School Tokyo Japan; ^24^ Department of Cardiology Keio University School of Medicine Tokyo Japan

**Keywords:** heart failure, hemodynamics, MitraClip, valvular disease, Valvular Heart Disease

## Abstract

**Background:**

Given the relatively high morbidity and death associated with transcatheter edge‐to‐edge repair for secondary mitral regurgitation, the development of optimal risk stratification models is imperative. The J‐MACS (Japanese Registry for Mechanically Assisted Circulatory Support) score is a recently developed tool designed to stratify risk in patients with advanced heart failure undergoing durable left ventricular assist device implantation.

**Methods:**

Data were obtained from the OCEAN‐Mitral (Optimized Catheter Valvular Intervention–Mitral) registry on patients with secondary mitral regurgitation and left ventricular ejection fraction <50% who underwent transcatheter edge‐to‐edge repair. A newly innovated modified J‐MACS score, incorporating age, history of cardiac surgery, serum creatinine levels, and postprocedural moderate or greater tricuspid regurgitation, was calculated. Its prognostic significance regarding the primary outcome, comprising all‐cause death and heart failure–related hospitalizations, was assessed.

**Results:**

A total of 2006 patients (median age, 77 years; 63% men) were included in the study. The median modified J‐MACS score was 13.7 (interquartile range, 12.0–16.2). Based on statistically calculated optimal cutoff values of 11.4 and 14.0, patients were stratified into 3 risk categories: low, moderate, and high. The 2‐year cumulative incidence of the primary outcome differed significantly among these groups (26%, 37%, and 51%, respectively; *P*<0.001). Risk group classification remained independently associated with the primary outcome, with an adjusted hazard ratio of 1.42 (95% CI, 1.13–1.71; *P*<0.001) for the intermediate risk versus low risk and 2.27 (95% CI, 1.67–2.96; *P*<0.001) for the high risk versus low risk.

**Conclusions:**

The modified J‐MACS score demonstrated an independent association with all‐cause death and heart failure–related hospitalization following transcatheter edge‐to‐edge repair in patients with significant mitral regurgitation and systolic heart failure.

**Registration:**

URL: https://upload.umin.ac.jp; Unique Identifier: UMIN000023653.

**Nonstandard Abbreviations and Acronyms**
HFrEF: heart failure with reduced ejection fraction
J‐MACS: Japanese Registry for Mechanically Assisted Circulatory Support
mJ‐MACS score: modified Japanese Registry for Mechanically Assisted Circulatory Support score
MR: mitral regurgitation
OCEAN‐Mitral: Optimized Catheter Valvular Intervention–Mitral
TEER: transcatheter edge‐to‐edge repair

**Clinical Perspective**

**What is New?**

- We applied the J‐MACS (Japanese Registry for Mechanically Assisted Circulatory Support) score, originally developed to estimate clinical outcomes after durable left ventricular assist device implantation, to patients with systolic heart failure undergoing mitral transcatheter edge‐to‐edge repair, given their overlapping background.
- We modified the original score and established a novel model for predicting 2‐year clinical outcomes after mitral transcatheter edge‐to‐edge repair, including age, history of cardiac surgery, serum creatinine level, and the presence of moderate or greater tricuspid regurgitation after the procedure.

**What Are the Clinical Implications?**

- The modified J‐MACS score independently predicts 2‐year clinical outcomes following mitral transcatheter edge‐to‐edge repair and can aid in risk stratification beyond conventional clinical parameters; this simple and practical tool may help clinicians identify high‐risk patients in the postprocedural phase who could benefit from more intensive monitoring or aggressive medical optimization.

Transcatheter edge‐to‐edge repair (TEER) has emerged as a pivotal therapeutic strategy for patients with significant mitral regurgitation (MR), particularly those with heart failure with reduced ejection fraction who are unsuitable candidates for surgical intervention.^1^, ^2^, ^3^, ^4^ Despite advancements in TEER technology and refined patient selection criteria, long‐term clinical outcomes remain unsatisfactory.^5^ Optimizing patient selection and implementing appropriate management strategies before and after the procedures are crucial for further improving postprocedural morbidity and death.

Previous studies have identified several risk factors associated with adverse clinical outcomes following TEER. For instance, the presence of residual valvular diseases and pulmonary hypertension were associated with worse clinical outcomes following TEER.^6^, ^8^ Parameters associated with advanced heart failure, including the presence of right ventricular dysfunction and advanced New York Heart Association functional class were also associated with post‐TEER clinical outcomes.^9^, ^10^ Other studies identified several risk factors, including advanced age, chronic kidney disease, and malnutrition.^11^, ^12^, ^13^ However, a comprehensive risk‐stratification model has yet to be established. Given the complex pathophysiology of heart failure and secondary MR, a multifactorial scoring system incorporating several prognostic variables is likely to provide superior risk stratification compared with the single risk factor.

The J‐MACS (Japanese Registry for Mechanically Assisted Circulatory Support) score was recently innovated to stratify risk among candidates for durable left ventricular assist devices (LVADs) using data from the nationwide, multicenter J‐MACS registry.^14^ This score has demonstrated superior predictive accuracy compared with conventional models, such as the HeartMate II risk score, effectively identifying high‐risk LVAD candidates. Advanced heart failure is frequently accompanied by significant MR, and there is considerable overlap in the clinical characteristics of patients undergoing TEER and those receiving durable LVAD therapy.^15^ Accordingly, we hypothesized that the J‐MACS score may also serve as a valuable prognostic tool in patients with systolic heart failure and secondary MR receiving TEER.

In this study, we aimed to assess the applicability of the J‐MACS score in predicting clinical outcomes among patients with systolic heart failure and secondary MR who underwent TEER, using data from the multicenter OCEAN‐Mitral (Optimized Catheter Valvular Intervention–Mitral) registry.

## Methods

### Participant Selection

The data that support the findings of this study are available from the corresponding author upon reasonable request. This retrospective study was conducted using a prospectively developed, multicenter, investigator‐driven OCEAN‐Mitral registry data set.^5^ The registry collected clinical data on patients who underwent TEER using the MitraClip system for significant MR between April 2018 and June 2023.

From this cohort, we excluded patients with primary MR, those with left ventricular ejection fraction (LVEF) of ≥50%, those dependent on hemodialysis, and those with critical data deficiencies. As a result, we included only the individuals with secondary ventricular MR. The study was registered with the University Hospital Medical Information Network Clinical Trials Registry (UMIN000023653). The research protocol for the creation of the registry database received approval from the institutional review board at each participating hospital, in compliance with the ethical principles outlined in the Declaration of Helsinki. All authors provided informed consents before the listing on the registry.

### Study Design

Patients were followed for a period of 2 years after undergoing TEER or until the termination of the observation period, with day 0 defined as the date of the TEER procedure. The independent variable was a modified J‐MACS (mJ‐MACS) score,^14^ a risk score as detailed below. The primary outcome was a composite of all‐cause death and heart failure–related hospitalizations following TEER. Hospitalization for worsening heart failure, necessitating intravenous diuretics or other related therapies during careful in‐hospital monitoring, was determined at the discretion of the attending board‐certified cardiologists.

### Calculation of Modified J‐MACS Score

The J‐MACS score was originally developed to estimate the 3‐year mortality rate following implantation of durable LVAD by using a nationwide J‐MACS registry.^14^ The J‐MACS score is calculated using 4 terms: 0.105×(age [years])+2.06×(history of cardiac surgery)+3.56×(serum creatinine (mg/dL])+2.61×(ratio between central venous pressure and pulmonary artery wedge pressure>0.71).

The OCEAN‐Mitral registry does not include comprehensive right heart catheterization data before TEER because TEER was sometimes undergone without prior right heart catheterization.^5^ We could not calculate the original J‐MACS score. Instead, we replaced the right heart catheterization data with the presence of moderate or greater tricuspid regurgitation following TEER. Its impact on the formula was estimated by calculating the hazard ratio for the primary outcome. We constructed an mJ‐MACS score consisting of age, history of cardiac surgery, serum creatinine, and moderate or greater tricuspid regurgitation following TEER.

### TEER Procedure

The decision to proceed with TEER was made by multidisciplinary local heart‐valve teams comprising interventional cardiologists, general cardiologists, cardiothoracic surgeons, imaging cardiologists, medical engineers, and other skilled health care professionals. The severity of MR was quantified using the proximal isovelocity surface area method by board‐certified echocardiography experts at each institution. This decision‐making process involved comprehensive discussions with patients and their families, ensuring that informed consent procedures were rigorously followed.

The TEER procedure using the MitraClip system was conducted according to standardized protocols. It was performed under general anesthesia with fluoroscopic and transesophageal echocardiographic guidance. The procedure began with a transseptal puncture via femoral vein access, followed by advancing a 24‐Fr guide catheter into the left atrium. The clip delivery system was then positioned above the origin of the MR jet and further advanced into the left ventricular cavity. The mitral leaflets were engaged, and the clip was temporarily closed to approximate the leaflets. If a satisfactory reduction in MR was achieved, the clip was released. If additional MR reduction was necessary, a second clip was considered on the basis of an assessment of the residual MR and the mean pressure gradient across the mitral valve.

### Follow‐Up

Patients were closely monitored during the index hospitalization to detect any periprocedural complications. Discharge was considered after confirming the absence of critical procedure‐related complications. Following discharge, patients were regularly followed up at the outpatient clinic of each institution or their affiliated centers in a scheduled manner by board‐certified cardiologists. Heart failure medications were adjusted on the basis of the patient’s symptoms and test results obtained at each visit, at the discretion of the attending cardiologists.

### Clinical Variables Obtained

All data used in the present study were retrieved from the prefixed OCEAN‐Mitral registry database.^5^ Clinical variables included pre‐TEER characteristics, such as demographic information, comorbidities, laboratory findings, echocardiographic evaluations, and medication records. Echocardiographic assessments were conducted in accordance with the guidelines established by the American Society of Echocardiography.^16^

Procedural data, including anesthesia time, were also collected. Following the procedure, additional echocardiographic data and medication information were obtained. Notably, we did not have comprehensive laboratory data following TEER. Clinical outcomes were tracked for 2 years after the procedure or until the end of the study period (May 2024). The primary outcome was defined as a composite of all‐cause death and heart failure admissions during the observation period.

### Statistical Analyses

Continuous variables were presented as medians with interquartile ranges, while categorical variables were expressed as numbers and corresponding percentages.

The optimal cutoffs of the mJ‐MACS score to stratify the cumulative incidence of the primary outcome were determined using the minimum *P* value approach based on the log‐rank test. Consequently, the whole cohorts were divided into 3 risk groups using the calculated cutoffs: low‐risk group, intermediate‐risk group, and high‐risk group.

The cumulative incidence of the primary outcome was stratified by the 3 groups of mJ‐MACS score using Kaplan–Meier analysis and compared among them by the log‐rank test. The prognostic impact of the mJ‐MACS score on the primary outcome was assessed by Cox proportional hazard ratio regression analysis, together with other potential risk factors that were significant in the univariable analyses, following the confirmation that their variance inflation factors were <5.0. The model’s discriminative ability as a continuous variable was also evaluated with Harrell’s C‐index, applying bootstrap optimism‐correction with 1000 resamples. A restricted cubic spline analysis was also adopted to assess the predictability of mJ‐MACS score as a continuous variable, with 5 degrees of freedom.

All statistical analyses were conducted using SPSS Statistics software version 23.0 (IBM Corp, Armonk, NY). A 2‐sided *P* value of <0.05 was considered statistically significant for all tests.

## Results

### Patient Selection

Of the 3764 patients who underwent TEER and were registered in the OCEAN‐Mitral registry, 1125 (30%) patients with primary MR, 612 (16%) patients with an LVEF of ≥50%, and 21 (1%) patients with critical data deficiencies were excluded. Consequently, a total of 2006 patients with secondary MR and an LVEF of <50% (ie, secondary ventricular MR) were included in the present study.

### Clinical Data Before TEER

Baseline characteristics before TEER are displayed in Table 1. Median age was 77 (interquartile range, 70–83) years, and 1271 (63%) were men. Median EuroScore II was 6.1 (interquartile range, 3.8–10.6). 201 (10%) had histories of cardiac surgery. A total of 354 (18%) patients received continuous intravenous inotropes before TEER. Serum creatinine was 1.4 (1.0–2.0) mg/dL and plasma B‐type natriuretic peptide was 508 (249–970) pg/mL. LVEF was 32% (26%–38%) and the effective regurgitant orifice area of MR was 0.32 (0.24–0.43) cm^2^.

**Table 1:** Baseline Characteristics Before TEER

Demographics	
Age, y	77 (70–83)
Male sex	1271 (63)
Body mass index, kg/m^2^	21.1 (18.7–23.3)
Systolic blood pressure, mm Hg	101 (92–115)
Pulse rate, bpm	72 (64–83)
EuroScore II	6.1 (3.8–10.6)
New York Heart Association functional class I/II/III/IV	30/642/956/378
Comorbidity	
Hypertension	1218 (61)
Dyslipidemia	1113 (56)
Diabetes	687 (34)
History of major bleeding	115 (6)
Coronary artery disease	
Atrial fibrillation	1164 (58)
Peripheral artery disease	225 (11)
History of cardiac surgery	201 (10)
History of stroke	229 (11)
Chronic obstructive pulmonary disease	168 (8)
Intravenous inotropes use	354 (18)
Laboratory data	
Hemoglobin, g/dL	11.8 (10.6–13.2)
Serum albumin, g/dL	3.6 (3.2–4.0)
Serum total bilirubin, mg/dL	0.7 (0.5–1.0)
Serum creatinine, mg/dL	1.4 (1.0–2.0)
Plasma B‐type natriuretic peptide, pg/mL	508 (249–970)
Echocardiography data	
Left ventricular end‐diastolic diameter, cm	62 (56–68)
Left ventricular ejection fraction, %	32 (26–38)
Left atrial volume index, mL/m^2^	74 (58, 97)
MR effective regurgitant orifice area, cm^2^	0.32 (0.24–0.43)
Tricuspid regurgitant peak gradient, mm Hg	32 (23–42)
Moderate or greater aortic regurgitation	183 (9)
Moderate or greater tricuspid regurgitation	618 (31)
Medication	
β blocker	1671 (83)
Renin–angiotensin system inhibitor	1336 (67)
Mineralocorticoid receptor antagonist	1244 (62)
Diuretics	1634 (82)

Continuous variables are stated as median interquartile range) and categorical variables are stated as numbers (percentages). MR indicates mitral regurgitation; and TEER, transcatheter edge‐to‐edge repair.

### Clinical Data Following TEER

TEER was performed with an acute procedural success rate of 97%. Most patients received 1 clip implantation (1500 [75%]), and multiple clips were implanted in 498 (25%) patients.

Postprocedural echocardiography and medication data were displayed in Table 2. Moderate or greater MR following TEER was observed in 400 (20%) patients. Moderate or greater tricuspid regurgitation was observed in 452 (23%) patients.

**Table 2:** Post‐TEER Clinical Parameters

Procedure data	
Anesthesia time, min	146 (119–186)
Procedural time, min	79 (58–107)
Device implant time, min	51 (36–74)
Implanted clip number 0/1/2/3	8/1500/477/21
Acute procedural success	1945 (97)
Complications	
Pericardial effusion	60 (3)
Access site‐related complications	28 (1)
Transesophageal echocardiography–related complications	11 (1)
Acute kidney injury	50 (3)
Gastrointestinal bleeding	28 (1)
Other minor bleeding	12 (0.6)
Other major bleeding	16 (0.8)
Ischemic stroke	39 (2)
Hemorrhagic stroke	13 (0.6)
Transient ischemic attack	3 (0.1)
Infectious endocarditis	2 (0.1)
Repeated intervention on the mitral valve	50 (3)
Length of intensive care unit, d	1 (1–2)
Length of hospitalization, d	15 (8–30)
Echocardiography data	
Left ventricular end‐diastolic diameter, cm	60 (54–67)
Left ventricular ejection fraction, %	30 (25–37)
Left atrial volume index, mL/m^2^	69 (54–92)
Tricuspid regurgitation peak gradient, mm Hg	27 (22–34)
Moderate or greater atrial regurgitation	179 (9)
Moderate or greater MR	400 (20)
Moderate or greater tricuspid regurgitation	452 (23)
Medication	
β blocker	1305 (65)
Renin–angiotensin system inhibitor	715 (36)
Mineralocorticoid receptor antagonist	952 (48)
Diuretics	1174 (59)

Continuous variables are stated as median (25% interquartile range) and categorical variables are stated as numbers (percentages). MR indicates mitral regurgitation; and TEER, transcatheter edge‐to‐edge repair.

### Calculation of Modified J‐MACS Score

**Figure 1:**
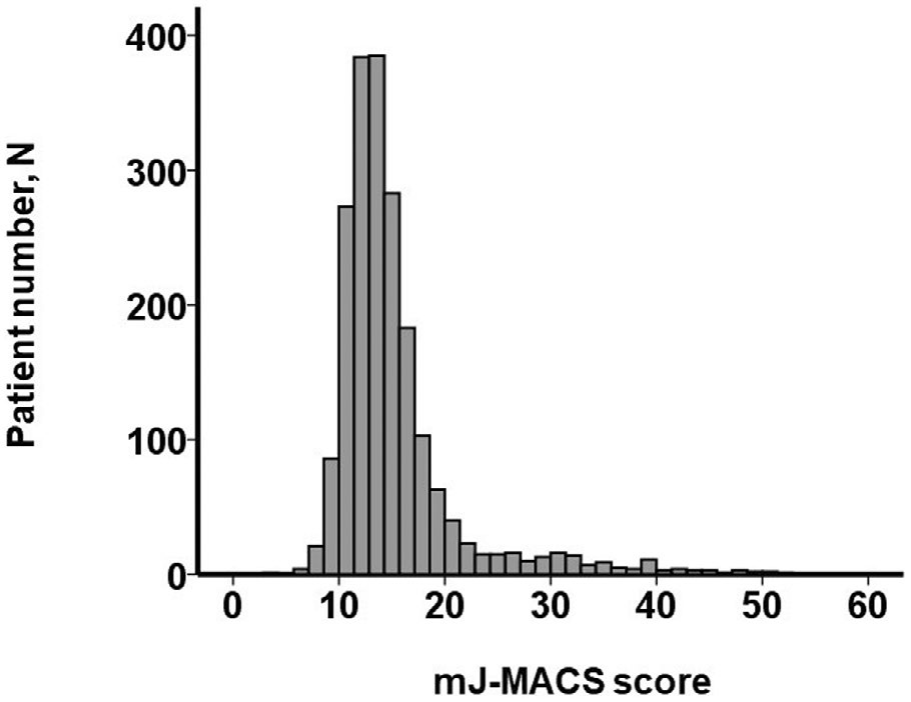
Distribution of mJ‐MACS score. mJ‐MACS score was distributed widely with nonnormal distribution pattern. mJ‐MACS indicates modified Japanese Registry for Mechanically Assisted Circulatory Support.

According to the hazard ratio of the presence of postprocedural moderate or greater tricuspid regurgitation for the primary outcome (1.40 [95% CI, 1.18–1.67]; *P*<0.001), we modified the conventional J‐MACS score by adding the term. Consequently, we constructed a modified formula, mJ‐MACS score, consisting of age, history of cardiac surgery, serum creatinine level, and the presence of post‐TEER moderate or greater tricuspid regurgitation: 0.105×(age [years])+2.06×(history of cardiac surgery)+3.56×(serum creatinine [mg/dL])+1.40×(presence of moderate or greater tricuspid regurgitation). The mJ‐MACS score was distributed widely, with a median value of 13.7 (interquartile range, 12.0–16.2) (Figure 1).

### Stratification of the Primary Outcome by Modified J‐MACS Score

During a median 416 (interquartile range, 296–730) days following TEER, 647 patients encountered the primary outcomes (391 patients had heart failure admissions, and 397 patients expired). The mJ‐MACS score, as a continuous variable, was significantly associated with the primary outcome with an unadjusted hazard ratio (HR) of 1.03 (95% CI, 1.02–1.04; *P*<0.001) (Table 3). When we did not include a history of cardiac surgery for calculating the score, its C‐index was statistically not different from those of mJ‐MACS (0.72 versus 0.71, *P*=0.78). The C‐statistic for the mJ‐MACS score was numerically higher than the Society of Thoracic Surgeons score (0.63, *P*=0.12) and EuroScore II (0.60, *P*=0.057).

**Table 3:** Potential Risk Factors for the Primary Outcomes

	Univariable analysis		Multivariable analysis	
	Hazard ratio (95% CI)	*P* value	Hazard ratio (95% CI)	*P* value
mJ‐MACS score (as continuous variables)	1.03 (1.02–1.04)	<0.001*	…	…
mJ‐MACS score (as categorical variables)				
mJ‐MACS score intermediate‐risk vs low‐risk	1.60 (1.35–1.90)	<0.001*	1.42 (1.13–1.71)	<0.001*
mJ‐MACS score high‐risk vs low‐risk	2.39 (1.88–3.05)	<0.001*	2.27 (1.67–2.96)	<0.001*
mJ‐MACS score breakdown				
Age	1.03 (1.02–1.03)	<0.001*	…	
History of cardiac surgery	0.96 (0.74–1.25)	0.77	…	
Serum creatinine, mg/dL (pre‐TEER)	1.08 (1.04–1.12)	<0.001*	…	
Moderate or greater tricuspid regurgitation (post‐TEER)	1.40 (1.18–1.67)	<0.001*	…	
Male sex	1.07 (0.91–1.26)	0.39	…	
Body mass index, kg/m^2^	0.97 (0.95–0.99)	0.009*	0.99 (0.97–1.03)	0.97
Atrial fibrillation (post‐TEER)	1.26 (1.08–1.48)	0.004*	1.42 (1.12–1.78)	0.003*
Intravenous inotropes (pre‐TEER)	1.65 (1.37–1.99)	<0.001*	1.43 (1.10–1.85)	0.008*
Hemoglobin, g/dL (pre‐TEER)	0.79 (0.76–0.83)	<0.001*	0.83 (0.77–0.88)	<0.001*
Common logarithm of plasma B‐type natriuretic peptide, pg/mL (pre‐TEER)	2.29 (1.86–2.80)	<0.001*	1.34 (1.03–1.74)	0.032*
Left ventricular ejection fraction, % (post‐TEER)	0.99 (0.98–0.99)	0.0038*	0.99 (0.98–1.01)	0.10
Residual moderate or greater MR (post‐TEER)	1.12 (0.93–1.34)	0.25	…	
β blocker (post‐TEER)	0.67 (0.57–0.78)	<0.001*	0.73 (0.57–0.93)	0.012*
Renin‐angiotensin system inhibitor (post‐TEER)	0.70 (0.59–0.83)	<0.001*	0.92 (0.72–1.17)	0.48
Mineralocorticoid receptor antagonist (post‐TEER)	0.71 (0.61–0.84)	<0.001*	0.90 (0.70–1.15)	0.39

Potential risk factors for the primary outcome were evaluated for their prognostic impact by the univariable Cox proportional hazard ratio regression analyses. Variables significant in the univariable analyses were included in the multivariable analysis. mJ‐MACS indicates modified Japanese Registry for Mechanically Assisted Circulatory Support; MR, mitral regurgitation; and TEER, transcatheter edge‐to‐edge repair.

* *P*<0.05.

In the restricted cubic spline analysis treating the mJ‐MACS score as predictors, the spline‐based HR curve showed steeper risk increases at higher score levels (Figure S1).

**Figure 2:**
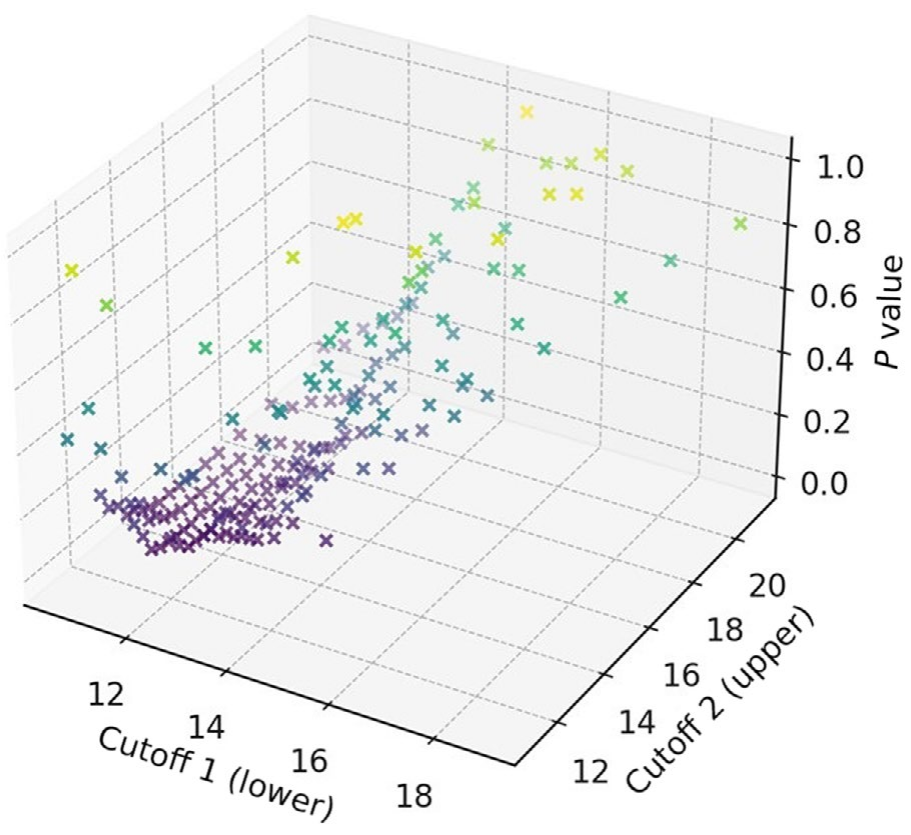
Calculation of optimal paired cutoffs of mJ‐MACS score to risk stratify the incidence of primary outcome. Optimal paired cutoffs of mJ‐MACS score (ie, a lower cutoff and an upper cutoff) with a minimum *P* value were sought to best stratify the risk of primary outcome by using the minimum *P* value approach based on the log‐rank test. A pair of 11.4 and 14.0 of mJ‐MACS score was eventually identified. mJ‐MACS indicates modified Japanese Registry for Mechanically Assisted Circulatory Support.

The Wilcoxon rank‐sum test across the mJ‐MACS scores demonstrated the optimal pair of cutoffs that had a minimum *P* value: 11.4 and 14.0 (Figure 2). The whole cohort was assigned to the 3 risk groups: low‐risk (mJ‐MACS score <11.4), intermediate‐risk (11.4 ≤mJ‐MACS score <14.0), and high‐risk groups (14.0 ≤mJ‐MACS score) according to the calculated cutoffs.

**Figure 3:**
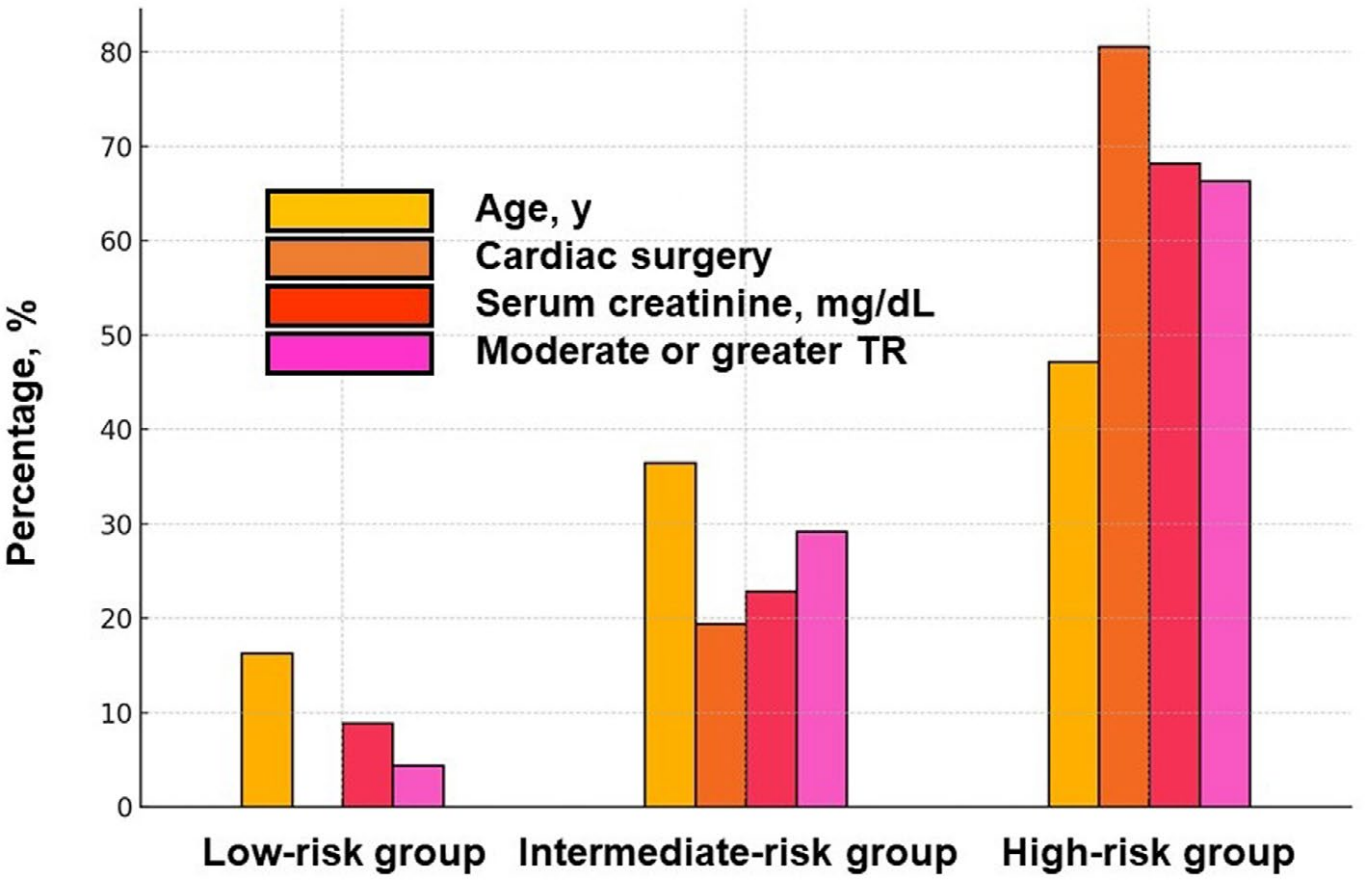
Percentage of each item of mJ‐MACS score among the three risk groups. Items consisted of age, history of cardiac surgery, serum creatinine, and moderate or greater tricuspid regurgitation following transcatheter edge‐to‐edge repair. TR indicates tricuspid regurgitation.

The percentages of each term (ie, age, history of cardiac surgery, serum creatinine, and moderate or greater tricuspid regurgitation) in the 3 groups are displayed in Figure 3. All 4 terms tended to be higher as incremental risks.

**Figure 4:**
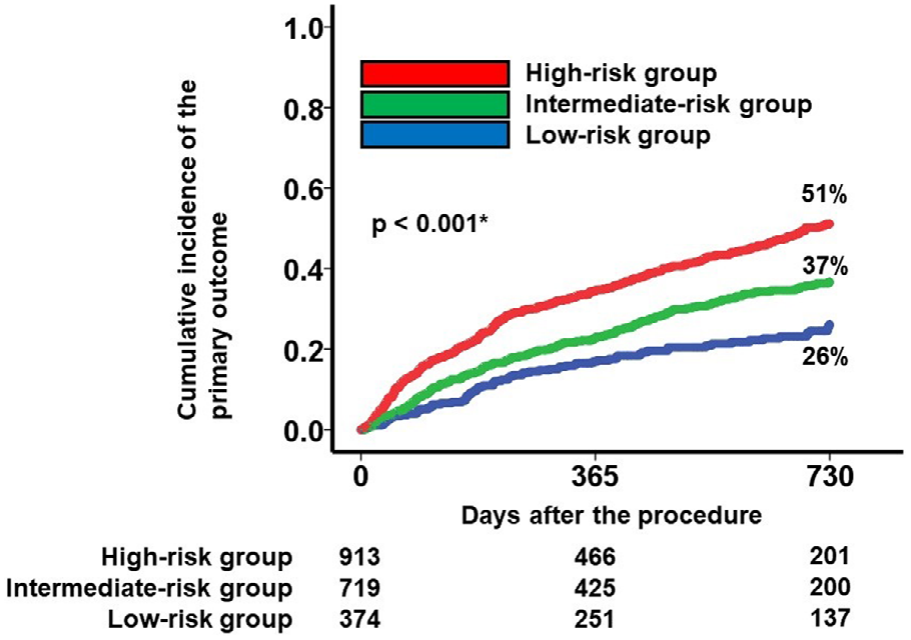
Prognostic impact of mJ‐MACS score group. Cumulative incidence of the primary outcome was stratified into 3 groups according to the mJ‐MACS score group. *P<0.05 by log‐rank test. mJ‐MACS indicates modified Japanese Registry for Mechanically Assisted Circulatory Support.

The mJ‐MACS score, which was divided into the 3 risk groups, significantly stratified the cumulative incidence of the primary outcome (26%, 37%, and 51% for the 2‐year cumulative incidence; *P*<0.001; Figure 4). As a breakdown of the outcome, the mJ‐MACS score significantly stratified the cumulative incidence of death (HR, 1.59 [95% CI, 1.38–1.84]; *P*<0.001) and heart failure admission (HR, 1.56 [95% CI, 1.40–1.75]; *P*<0.001), respectively.

Prognostic Impact of Modified J‐MACS Score Among Other Potential Risk Factors:

The prognostic impacts of the potential risk factors for the primary outcomes were preselected and listed in Table 3. As a breakdown of mJ‐MACS score, age, serum creatinine, and moderate or greater tricuspid regurgitation post‐TEER were significantly associated with the primary outcome.

After adjusting for other significant univariable predictors, the mJ‐MACS score remained independently associated with the primary outcomes for the intermediate‐risk versus low‐risk (adjusted HR, 1.42 [95% CI, 1.13–1.71]; *P*<0.001) groups and for the high‐risk versus low‐risk (adjusted HR, 2.27 [95% CI, 1.67–2.96]; *P*< 0.001), respectively. Additional independent predictors included atrial fibrillation, inotrope use, hemoglobin level, B‐type natriuretic peptide concentration, and β blocker use.

## Discussion

In this retrospective analysis, we assessed the prognostic utility of the mJ‐MACS score, originally developed to stratify risk among patients undergoing durable LVAD implantation, in a cohort of patients with systolic heart failure and secondary MR treated with TEER, using data from the prospectively collected, multicenter OCEAN‐Mitral registry.^5^

The original J‐MACS score is composed of 4 readily obtainable variables: age, history of cardiac surgery, serum creatinine level, and invasively measured right ventricular function data.^14^ Due to the absence of comprehensive right heart catheterization data in the registry,^5^ we substituted this parameter with the presence of moderate or greater tricuspid regurgitation following TEER, an echocardiographic practical surrogate reflecting right ventricular dysfunction. Importantly, we elected to use postprocedural rather than preprocedural TR data, as the former demonstrated stronger prognostic relevance.^6^

Our findings indicate that the newly innovated mJ‐MACS score serves as an independent predictor of 2‐year all‐cause death and heart failure rehospitalization following TEER. It retained significance alongside several other established prognostic variables.

### Rationale for Focusing on J‐MACS Score

Candidates for durable LVADs frequently present with significant secondary MR, resulting from adverse left ventricular remodeling and mitral valve tethering. Notably, this form of MR can often be ameliorated solely through LVAD implantation, owing to hemodynamic unloading of the left ventricle, without direct intervention on the mitral valve itself.^15^ Reflecting this phenomenon, the presence or severity of baseline MR was not incorporated into the original J‐MACS score, although many LVAD candidates have significant MR at baseline.^14^

In the present study, we analyzed a cohort of patients with secondary MR and heart failure with reduced ejection fraction, a clinical profile that closely resembles that of typical LVAD candidates.^14^ In fact, the choice between TEER and LVAD implantation as an optimal interventional strategy is often debated on a case‐by‐case basis in this population. Similar to LVAD therapy, successful TEER frequently results in substantial reduction of MR severity and improvement of cardiac function.^5^

Given these overlapping clinical characteristics and shared pathophysiological mechanisms, we postulated that the J‐MACS score, although originally designed for LVAD risk stratification, may also hold prognostic value in patients undergoing TEER.

### Modification of J‐MACS Score

The original J‐MACS score incorporates an invasively measured hemodynamic parameter—the ratio of pulmonary artery wedge pressure to central venous pressure—as a surrogate for right ventricular function.^14^ Similar to other risk stratification models for LVAD candidates,^17^, ^18^ invasive hemodynamic indices have also been shown to predict long‐term outcomes in patients undergoing TEER. For instance, elevated mean pulmonary artery pressure and increased pulmonary vascular resistance have been associated with adverse post‐TEER prognosis.^7^

However, right heart catheterization is not routinely performed in TEER candidates, and comprehensive invasive data are often unavailable in real‐world clinical practice. To enhance the score’s practicality and generalizability, we replaced the invasive parameter with the presence of moderate or greater tricuspid regurgitation, a noninvasively assessed marker obtainable via transthoracic echocardiography.

This decision was further supported by recent evidence indicating that post‐TEER, rather than pre‐TEER, moderate or greater tricuspid regurgitation was more strongly associated with clinical outcomes.^6^ Given the intricate hemodynamic interplay between valvular pathologies, improvement of MR through TEER can reduce pulmonary vasculature pressures, subsequently lowering right ventricular afterload and alleviating functional tricuspid regurgitation,^19^ particularly in patients with low pulmonary vascular resistance. Consequently, baseline valvular abnormalities may not fully capture the dynamic changes that influence postprocedural outcomes.

Interestingly, a history of cardiac surgery, which is a key predictor in the LVAD setting,^14^ was not independently associated with outcomes in the TEER cohort. This discrepancy may be attributed to the different procedural invasiveness: while previous sternotomy confers increased risk for surgical reintervention like LVAD implantation, it appears to carry less prognostic significance in the context of catheter‐based, minimally invasive therapies such as TEER.

### How to Use the mJ‐MACS Score

It is important to emphasize that the mJ‐MACS score is not intended as a tool to exclude patients from receiving mitral TEER, especially given that patients with symptomatic secondary MR and reduced LVEF often have limited therapeutic options. Instead, the score aims to support clinicians in the postprocedural phase, helping to identify patients who may require closer monitoring or more aggressive medical optimization.

The mJ‐MACS score can be easily calculated using four straightforward variables—age, history of cardiac surgery, serum creatinine level, and postprocedural tricuspid regurgitation—all of which are obtainable noninvasively in the periprocedural period. The present findings underscore that even in cases of technically successful TEER, vigilant postprocedural management remains critical, particularly among high‐risk individuals. In such patients, procedural success alone, manifested by MR reduction and the absence of procedural complications, may not suffice to ensure favorable long‐term outcomes.

This aligns with growing evidence supporting a multidisciplinary care strategy surrounding TEER.^20^ High‐risk patients present with impaired right ventricular function, secondary pulmonary hypertension, and renal dysfunction. In this context, careful monitoring of systemic and pulmonary congestion, along with tailored adjustment of diuretic therapy, may be essential to optimize outcomes. Furthermore, by improving hemodynamic stability, TEER may facilitate the initiation or uptitration of guideline‐directed medical therapies that were previously intolerable due to circulatory compromise.^21^ Optimization of renoprotective agents may improve renal function and clinical outcomes. The catheter intervention for residual tricuspid regurgitation may stabilize hemodynamics and improve clinical outcomes.

While durable LVAD implantation is typically preferred for patients with profoundly remodeled ventricles and severe secondary MR, caution is warranted when considering this strategy in high‐risk individuals. Prior cardiac surgery increases procedural complexity due to the need for repeat sternotomy, and baseline right ventricular dysfunction may deteriorate after LVAD implantation as a result of interventricular septal shift, right heart remodeling, and increased venous return.^22^, ^23^

Several other clinical variables, including atrial fibrillation, inotrope use, anemia, elevated plasma B‐type natriuretic peptide levels, and β blocker therapy, were independently associated with adverse outcomes in this study. Although future research is warranted to develop an integrated risk model that incorporates these parameters, the practical utility of such models must be carefully balanced. While multifactorial scores may improve predictive accuracy, excessive complexity may limit their adoption in real‐world clinical settings.

### Limitations

While the predictive power of clinical judgment remains essential, structured tools such as the mJ‐MACS score offer standardized, reproducible means of risk assessment, which is especially valuable in multicenter and diverse clinical environments. The mJ‐MACS score was not developed to replace clinical experience but rather to aid in postprocedural risk stratification. Because the mJ‐MACS score includes a postprocedural parameter, it is not suitable for preprocedural risk stratification or patient selection. Instead, it should be interpreted as a postinterventional risk assessment tool to guide follow‐up intensity and adjunctive therapy. We included only the individuals with secondary ventricular MR with LVEF <50%, because the original J‐MACS score was developed for heart failure with reduced ejection fraction cohort. Thus, the applicability of our findings to individuals with atrial functional MR and those with primary MR remains uncertain.

We substituted tricuspid regurgitation for the invasive right heart catheterization parameter included in the original J‐MACS score. While tricuspid regurgitation was selected as a noninvasive and practical surrogate marker of right heart dysfunction, the validity and prognostic equivalence of this substitution require further validation.

Although prior cardiac surgery was not a statistically significant predictor in the present study, we retained this variable in the mJ‐MACS score to preserve structural consistency with the original J‐MACS model and to allow for future comparative validation. We believe that the post hoc modification of the items in the scoring following the completion of statistical analysis is unfair. Additionally, in selected TEER cases, prior cardiac surgery may still pose clinical implications due to altered cardiac anatomy or comorbid procedural complexity, even in the absence of sternotomy.

The OCEAN‐Mitral registry did not include laboratory data obtained immediately following TEER.^5^ Consequently, in calculating the mJ‐MACS score, we used preprocedural serum creatinine values, which may not fully capture renal function changes induced by the intervention.

Echocardiographic data, including MR severity assessments, were not analyzed by an independent core laboratory. Furthermore, comprehensive echocardiographic evaluation of right ventricular function, which may play a pivotal role in post‐TEER prognosis, was not consistently available. This lack of standardized imaging data represents an additional limitation of the study.

## Conclusions

The mJ‐MACS score demonstrated an independent association with all‐cause death and heart failure–related hospitalization following TEER in patients with significant MR and systolic heart failure. This simple, noninvasive risk stratification tool may aid in identifying high‐risk patients and optimizing postprocedural management strategies.

## Sources of Funding

The OCEAN‐Mitral registry, which is part of the Optimized Catheter Valvular Intervention–Structural Heart Disease registry, is supported by Edwards Lifesciences, Medtronic Japan, Boston Scientific Japan, Abbott Medical Japan, and Daiichi‐Sankyo Company.

## Disclosures

Drs Ueno, Kubo, Izumi, Saji, Izumo, Watanabe, Amaki, and Kodama are clinical proctors of TEER for Abbott Medical and have received consultant fee from Abbott Medical. Drs Asami and Kodama are clinical proctors of TEER for Abbott Medical and have received speaker fees from Abbott Medical. Dr Yamamoto is a clinical proctor of TEER for Abbott Medical and has received a lecture fee from Abbott Medical. Dr Otsuki has received a lecture fee and a scholarship donation from Abbott Medical. Dr Ohno has received consultant, advisor, and speaker fees from Abbott Medical. Drs Enta, Shirai, Mizuno, and Bota are clinical proctors of TEER for Abbott Medical. The remaining authors have no disclosures to report.

## Supporting information

OCEAN‐Mitral investigators list
Figure S1
