# Supplementary material for: Prognostic Impact of Modified J‐MACS Score in Patients With Systolic Heart Failure Receiving Transcatheter Edge‐to‐Edge Mitral Valve Repair
Source: J Am Heart Assoc. 2025 Sep 30;14(19):e043819. doi: 10.1161/JAHA.125.043819 (PMC12684534; doi:10.1161/JAHA.125.043819)
Supplement: Supplementary file 1 — OCEAN‐Mitral investigators list Figure S1 [file JAH3-14-e043819-s001.pdf]

SUPPLEMENTAL MATERIAL

OCEAN-Mitral investigators list

Keio University
Kentaro HAYASHIDA, Makoto TANAKA, Hikaru TSURUTA, Tetsuya SAITO, Yusuke KOBARI, Toshinobu RYUZAKI, Tatsuo TAKAHASHI, Shinichi GOTO, Shohei IMAEDA, Shingo SAKATA, Yoshinori KATSUMATA, Kohsuke SHIRAKAWA, Juri IWATA, Keitaro SHINADA, Akiyoshi KAJINO, Jungo KATO, Ryo ARITA, Tomonari MORIIZUMI

Toyohashi Heart Center
Masanori YAMAMOTO, Mitsuru SAGO, Tatsuya TSUNAKI, Ryo YAMAGUCHI, Junji YANAGISAWA, Yuki OKUBO

Nagoya Heart Center
Ai KAGASE, Takahiro TOKUDA, Yuki NAKASHIMA, Toshihiro KOBAYASHI, Kenichi SHIBATA, Ryoutaku KAWAHATA, Hiroto NISHIO, Yuki KONDO, Tomofumi NAKAMURA, Hiroshi TSUNAMOTO

Gifu Heart Center
Tetsuro SHIMURA, Yutaka KOYAMA, Munenori OKUBO, Shunsuke IMAI, Tokuya SAKAKURA, Yoshihiro HARANO, Masahiro INAGAKI, Hirooki HIGAMI, Yoji KUZE, Takashi NAGAI, Azusa KURITA

Kokura Memorial Hospital
Shinichi SHIRAI, Akihiro ISOTANI, Kenichi ISHIZU, Masato FUKUNAGA, Hiroyuki TABATA, Toru MOROFUJI,Hiroyuki KONO, Maiko KURODA, Euihong KO, Norihisa MIYAWAKI, Kenji NAKANO, Katsunori MIYAHARA, Ko YAMAMOTO, Koumei ONUKI, Yasuo TSURU, Tomohiro SUENAGA, Akira OTANI

Teikyo University
Yusuke WATANABE, Taiga KATAYAMA, Hirofumi HIOKI, Junichi NISHIKAWA, Yosei ISEKI, Yasuyuki TSUCHIDA, Kento KITO, Joe OMIYA, Masataka ARAKAWA, Maki OKAMOTO, Masafumi SAITO, Mizuki MIURA

New Tokyo Hospital
Toru NAGANUMA, Toru OUCHI, Haruhito YUKI

Saiseikai Yokohama City Eastern Hospital
Masahiro YAMAWAKI

Sendai Kousei Hospital
Yusuke ENTA, Norio TADA, Masaki NAKASHIMA, Makoto SAIGAN, Yoshiko MUNEHISA, Masaki MIYASAKA, Natsuko SATOMI, Yuta KOBAYASHI, Daishi TAZAWA, Yun TENG

Shonan Kamakura General Hospital
Shingo MIZUNO, Futoshi YAMANAKA, Koki SHISHIDO, Tomoki OCHIAI, Tsuyoshi YAMABE, Noriaki MORIYAMA, Takashi MATSUMOTO, Hirokazu MIYASHITA, Yoichi SUGIYAMA, Kunihiko SHIMIZU, Daisuke SATO, Yuka MURAKAMI

Toyama University Hospital
Hiroshi UENO, Nobuyuki FUKUDA, Shuhei TANAKA, Hiroshi ONODA, Toshio DOI, Mitsuo SOBAJIMA, Yohei UENO, Teruhiko IMAMURA, Ryuichi USHIJIMA, Yuki HIDA, Kazuya NAKASHIMA

Tokai University
Yohei OHNO, Junichi MIYAMOTO, Tsutomu MURAKAMI, Sho TORII
Makoto NATSUMEDA, Shigemitsu TANAKA, Takeshi IJICHI, Hitomi HORINOUCHI, Kazuki AIHARA, Katsuaki SAKAI, Shingo MATSUMOTO, Norihiko KAMIOKA, Norihito NAKAMURA, Satoshi NODA, Yu SATO

St. Marianna University School of Medicine
Masaki IZUMO, Daisuke MIYAHARA, Shingo KUWATA, Masashi KOGA, Toshiki KAIHARA, Takumi HIGUMA, Kei HONDA, Yoshikuni KOBAYASHI, Risako MURATA, Kenichi SASAKI, Daisuke TOGASHI, Takahiko KAI, Taishi OKUNO, Toshiya YOSHIDA, Keita SONE, Karin TERAUCHI

Sapporo Higashi Tokushukai Hospital
Hiroki BOTA, Kazumasa YAMASAKI, Tomoyuki TANI, Mamoru MIYAZAKI, Yuki KATAGIRI

Saiseikai Kumamoto Hospital
Kazuhisa KODAMA, Hiroto SUZUYAMA, Hideharu OKAMATSU, Tomohiro SAKAMOTO, Yoko HORIBATA, Yutaka KONAMI, Masahiro YAMADA, Eiji HORIO, Mika MAEDA, Satoko HASEGAWA, Megumi KOGA, Mikiko OHARA, Shota FUKUSHIGE

Tokyo Women's Medical University
Junichi YAMAGUCHI, Hisao OTSUKI, Yusuke INAGAKI, Chihiro KOYANAGI, Tomohito KOGURE, Takanori KAWAMOTO, Masafumi YOSHIKAWA, Eiji SHIBAHASHI, Kazuki TANAKA

Kurashiki Central Hospital
Shunsuke KUBO, Mikitaka FUJITA, Sachiyo ONO, Kohei OSAKADA, Kazunori MUSHIAKE, Takeshi MARUO, Naoki NISHIURA

National Cerebral and Cardiovascular Center
Makoto AMAKI, Kensuke TAKAGI, Takeshi KITAI, Atsushi OKADA, Tasuku HADA, Shoko NAKAGAWA, Marina ARAI, Yuki IRIE, Takahiro SAKAMOTO, Shinichi KURASHIMA

Mitsui Memorial Hospital
Masahiko ASAMI, Jun TANAKA, Yu HORIUCHI, Naoki HOSODA, Hideaki NONAKA, Yuki GONDA, Yohei NISHIMURA

Sakakibara Heart Institute
Mike SAJI, Yuki IZUMI, Ryosuke HIGUCHI, Ryo ABE, Takashi HIRUMA, Satonori MAEKAWARA, Koya OKABE, Yoshiko NEMOTO

Kindai University
Gaku NAKAZAWA, Masakazu YASUDA, Kosuke FUJITA, Tatsuya MIYOSHI, Naoko SOEJIMA, Masahiro MARUYAMA, Motohide TANAKA, Takayuki KAWAMURA, Honoka KANNO, Ayano YOSHIDA, Kyohei ONISHI, Nobuhiro YAMADA

Juntendo University Hospital
Shinya OKAZAKI, Minoru TABATA, Shinichiro DOI, Ryota NISHIO, Nobuyuki KAGIYAMA, Tomohiro KANEKO, Sakiko MIYAZAKI, Norihito TAKAHASHI

Sapporo Heart Center Sapporo Cardiovascular Clinic
Daisuke HACHINOHE, Ken KOBAYASHI, , Kazuki MIZUTANI, Hidemasa SHITAN, Ryo HORITA, Ryo OTAKE

Toho University Omori Medical Center
Mike SAJI, Hiroshi OHARA, Hideki KOIKE

Figure S1. Restricted cubic spline analysis for the mJ-MACS score

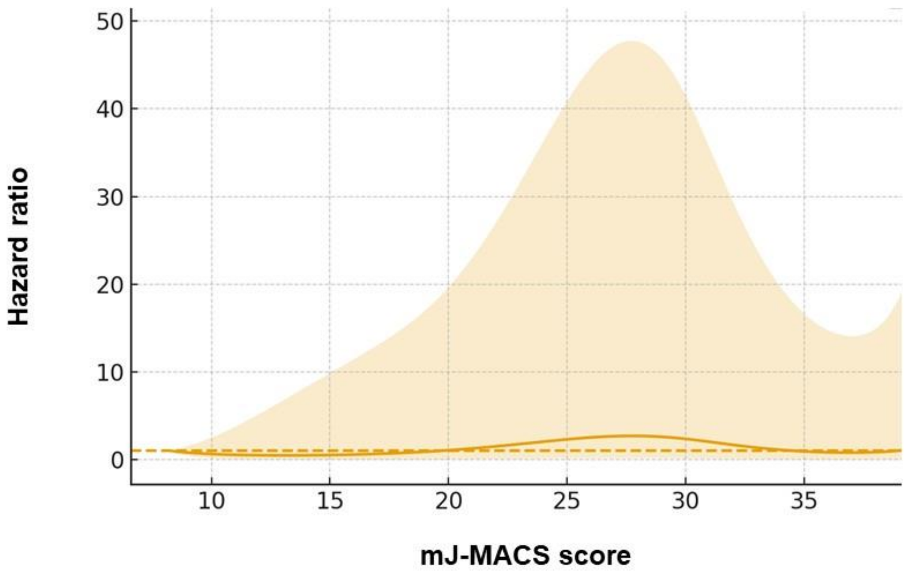

Restricted cubic spline functions with 5 degrees of freedom were used in a Cox proportional hazards model to flexibly model the association between mJ-MACS score and the risk of the endpoint. The solid line indicates the hazard ratio relative to the median score, and the shaded area indicates the 95% confidence interval.
